# A Rare Case of Congenital Ranula in an Infant

**DOI:** 10.1155/2016/5874595

**Published:** 2016-05-22

**Authors:** Sirin Mneimneh, Randa Barazi, Mariam Rajab

**Affiliations:** ^1^Pediatric Department, Makassed General Hospital, P.O. Box 11-6301, Beirut, Lebanon; ^2^Otolaryngology Department, American University of Beirut Medical Center, Beirut, Lebanon

## Abstract

Ranula is a mucus extravasation cyst originating from the sublingual gland on the floor of the mouth. Congenital ranula is very rare. We report a case of a 4-month-old girl with a congenital ranula in the floor of mouth. The ranula was treated first by marsupialization, but the cyst recurred after 1 week. Excision of the ranula was done and was successful.

## 1. Introduction

Congenital ranulas are rare epithelial lined retention cysts or pseudocysts, which arise in the floor of the mouth. The term ranula itself is derived from the Latin word* rana*, meaning frog, and ranula describing a little frog, denoting its resemblance to a bulging frog's underbelly [1]. They are thought to occur following obstruction of the main sublingual duct or acini causing extravasation of mucus into the surrounding tissues [2]. Most ranulas are asymptomatic. Treatment options include needle aspiration, surgical excision of the cyst, and sublingual gland excision along with the cyst, marsupialization, sclerotherapy, laser excision, or cryosurgery.

We describe a case of large congenital sublingual ranula associated with feeding difficulties and its way of management.

## 2. Case Report

**Figure 1:**
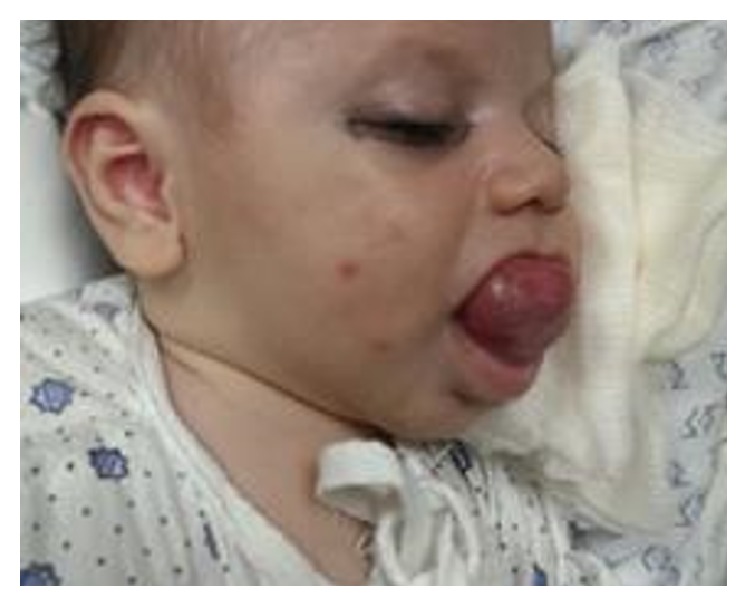
Large protruded tongue, showing a swelling in the floor of the mouth.

**Figure 2:**
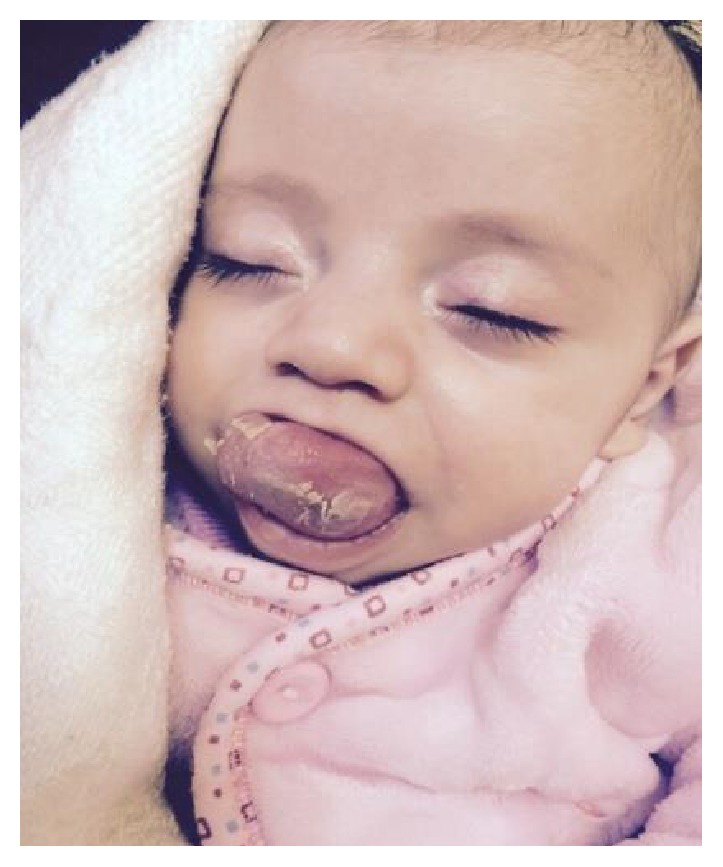
Recurrence of the swelling after the marsupialization.

A 4-month-old girl infant, previously healthy, presented with swelling in the floor of the mouth that was noticed immediately after birth. The swelling had increased in size over time causing difficulty in feeding. She had no associated pain. Clinical examination revealed a healthy-looking girl infant. She had a large protruded tongue, with a cystic swelling in the floor of the mouth measuring around 4 × 3 cm, with elevation of the tongue (Figure 1); she had no palpable neck masses and the tongue mobility was normal. At the age of 3 months, marsupialization was done, but one week later the swelling recurred with the same size (Figure 2).

**Figure 3:**
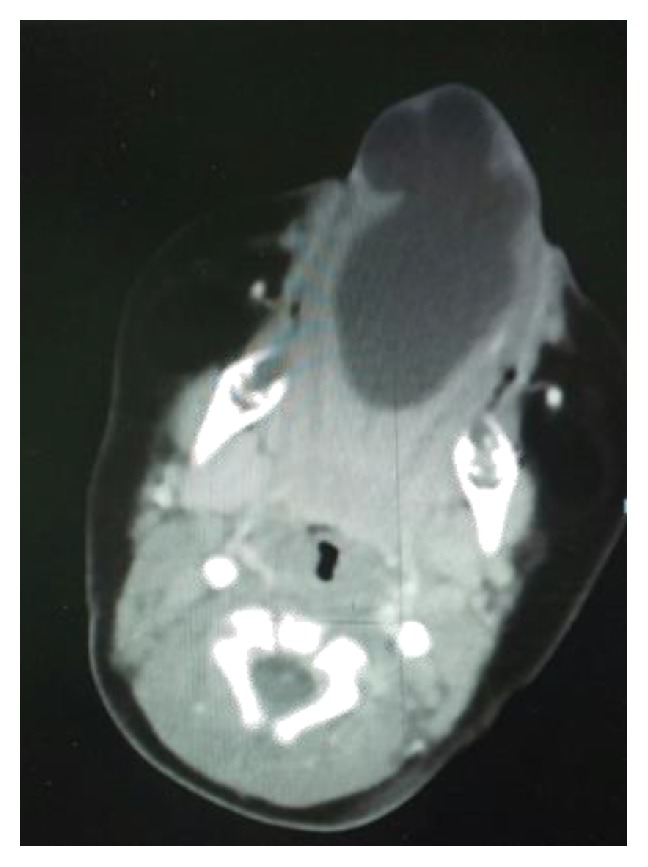
CT neck showing a 4 × 3 × 2 cm cyst in the floor of the mouth.

**Figure 4:**
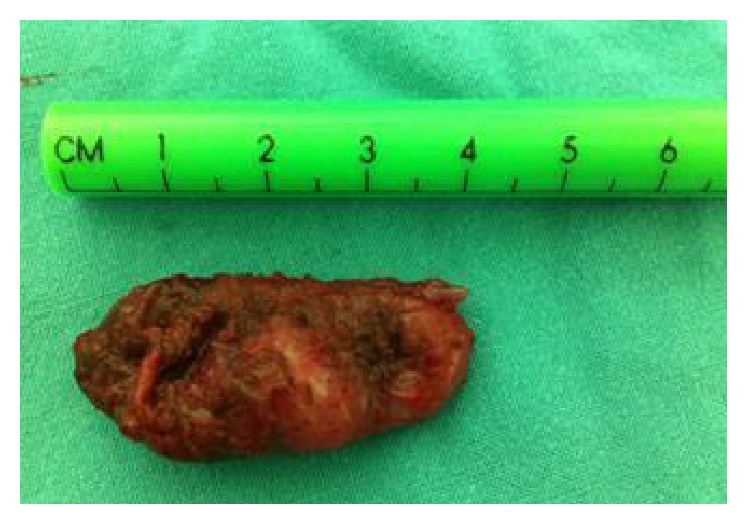
The cyst excised measures 4 × 2 cm.

**Figure 5:**
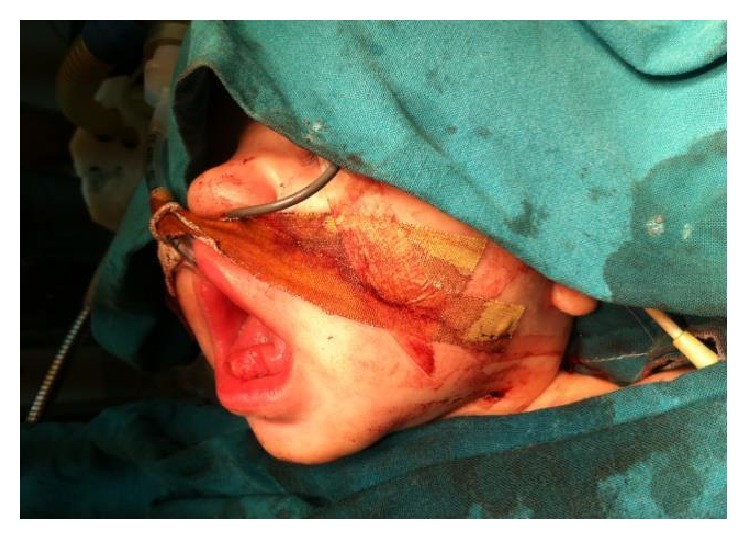
Marked reduction in the size of the mouth after excision of the cyst.

CT scan of the neck revealed a 4 × 3 × 2 cm cyst in the floor of the mouth, most consistent with ranula. Epidermoid and dermoid cysts were rare possibilities. There were no other masses, collection, or abnormal calcification (Figure 3). Decision was made to proceed with intraoral excision of the cyst. At 4 months of age surgical excision of the cyst was done with preservation of Wharton's ducts and the lingual nerves bilaterally that revealed a cyst of 4 × 2 cm (Figure 4), with marked reduction in the size of the tongue (Figure 5).

**Figure 6:**
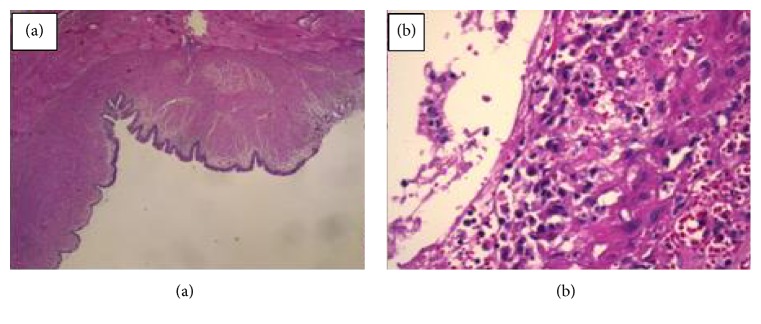
(a) Histopathology of the cyst. (b) High magnification view of the lining epithelium.

The histopathology showed a cyst lined by focally eroded columnar epithelium partly replaced by granulation tissue; it is composed of smooth muscle and surrounded by skeletal muscle, a pattern consistent with ranula (Figure 6).

## 3. Discussion

Ranulas are classified anatomically into 2 types: intraoral or simple ranulas which are confined to the sublingual space while cervical or plunging ranulas extend around the mylohyoid into the submandibular neck space [2]. Epidemiological data is limited, but the estimated overall prevalence of ranulas in the paediatric age group is 0.2 cases per 1000 [3]. Studies have shown that that oral ranulas peak in their frequency in the second decade of life [3]. Congenital ranulas are rare, with an incidence of 0.74%, with prenatal diagnosis rarely reported [4]. Congenital ranula is usually asymptomatic and resolves with time. Nevertheless, our patient had a large and symptomatic congenital ranula.

A prospective study evaluated ranula pathogenesis according to anatomical variation of the sublingual gland [4]. Usually numerous ductules from the posterior sublingual gland open onto the summit of the sublingual fold; however, several of the ductules can also join to form a common duct (Bartholin's duct) that empties into Wharton's duct. Through meticulous dissection, they found that 88.9% of simple ranulas contained Bartholin's duct in comparison to 42.9% of plunging ranulas. This was compared to 0% in control patients.

Ranulas are usually painless, fluctuant, with a blue translucent color swelling, and slowly growing mass of the floor of the mouth [5]. The diagnosis of ranula is made generally based on the clinical examination. Ultrasonography, computed tomography scanning, and magnetic resonance imaging can be helpful in determining the size and the location of the lesion. An MRI scan is considered a gold standard; besides giving high resolution images, it determines the precise location and content of the lesion and enhances the differentiation of ductal atresia from duplication anomalies of ductal system [6]. A fine-needle aspiration biopsy shows the mucus with inflammatory cells; in addition the biochemical analysis of aspiration fluid reveals high protein and amylase contents. Interestingly congenital lesions can now be diagnosed prenatally by ultrasonography and an EXIT (exuterointrapartum treatment) procedure may be followed for treatment in such cases [7, 8].

Management is variable and can range from aspiration to surgical excision. Treatment includes aspiration of mucus, incision and drainage, marsupialization, injection of sclerosing agents, excision of the ranula with or without excision of the ipsilateral sublingual gland, and CO_2_ laser excision [6–13].

In a review of 580 ranulas, recurrence rates for marsupialization, excision of ranula, and excision of the sublingual gland combined with the lesion were 66.7%, 57.7%, and 1.2%, respectively [3]. Besides minimizing the risk of recurrence, morbidity of sublingual sialoadenectomy is relatively high with risk of injury to Wharton's duct (2%), bleeding (1-2%), infection (1-2%), and lingual nerve paraesthesia (2–12%) [14].

The marsupialization is associated with high rate of recurrence [3]. Excision of the ranula with sublingual gland is the most suitable and effective surgical method for simple ranulas. The complications of surgery of ranula are intraoperative cyst rupture, injury to sublingual nerve and Wharton's duct, hemorrhage, and infection [13].

In summary, the rarity of this lesion in neonates and its atypical size make this case report unique. Finally excision of the ranula has lower recurrence rate compared to marsupialization so it is the appropriate treatment for congenital ranula.
